# Pregnancy in Teenage Romanian Mothers

**DOI:** 10.7759/cureus.21540

**Published:** 2022-01-24

**Authors:** Mihaela C Radu, Loredana S Manolescu, Razvan Chivu, Corneliu Zaharia, Calin Boeru, Melania-Elena Pop-Tudose, Andrei Necsulescu, Marina Otelea

**Affiliations:** 1 Department of Birth, Obstetrics and Gynecology Hospital, Ploiesti, ROU; 2 Virology, Microbiology and Parasitology, Carol Davila University of Medicine and Pharmacy, Bucharest, ROU; 3 Public Health and Health Management, Carol Davila University of Medicine and Pharmacy, Bucharest, ROU; 4 Biophysics Laboratory, Institute of Virology, Bucharest, ROU; 5 Obstetrics and Gynaecology, Carol Davila University of Medicine and Pharmacy, Bucharest, ROU; 6 Intensive Care Unit (ICU), Central Military Emergency University Hospital "Dr. Carol Davila”, Bucharest, ROU; 7 Public Health Sciences, Carol Davila University of Medicine and Pharmacy, Bucharest, ROU

**Keywords:** health public, risk factors, medical knowledge, mothers under 18 years of age, pregnancy

## Abstract

Background and purpose

Teenage pregnancy is associated with an increased risk of adverse pregnancy outcomes. The objective of this research is to determine the profile of the pregnant teenager and the medical complications associated with pregnancy at this young age.

Materials and methods

A cross-sectional study based on a 29-item questionnaire was conducted in 2019 and 2020 in Ploiești, Romania. The participants were divided into two groups, namely, Group A, consisting of 100 minor, teenage childbearing women under the age of 18, and Group B, consisting of 100 childbearing women over 18 years of age.

Results

Group A had a mean age of 16.56 ± 1.65. The percentage of births in very young girls (13-15 years) from group A is 28%. In 65 adolescents, sexual intercourse began at the age of 14. Pregnancy monitoring, expressed by the number of medical examinations, shows significant differences between the studied groups. The Short Assessment of Health Literacy (SAHL) test applied to both groups revealed a low level of health literacy in group A. Also in this group A, teenagers gave birth to low-birth-weight children, the percentage is statistically significant (14% vs. 4%). The gestational age in this group had an average of 36.88 ± 2.13 weeks, compared to the gestational age in the control group of 38.41 ± 1.57 weeks. In Romania, there are teenagers who became mothers at an early age. There should be educational programs in rural and urban schools and communities. Poverty leads to inadequate medical supervision with significant consequences for the health of the mother and child, lack of education (school dropout, illiteracy), and inability to find a job. The midwife can play a key role in rural communities through health education conducted on specific communication channels and with different forms of presentation of messages, adapted to their needs. A good target would be the parents of adolescent mothers and better communication with them.

## Introduction

The health of mothers and newborns are faithful indicators of the well-being of the general population. Pregnancy and parenting during adolescence (teenage) is a public health problem due to the high social and economic costs. Early school leaving due to pregnancy and birth prevents the completion of certain cycles of education for the acquisition of a profession, making it more difficult to find a job later. Unemployment and, consequently, poverty will affect this category of people. Promoting healthy pregnancy and safe births is a goal of all countries. The poor health of the mother and newborn has long-lasting consequences. The social context and consequences of these effects must also be considered, as the burden of poor health falls disproportionately on socially disadvantaged women and babies. Adverse perinatal health outcomes perpetuate health and social inequalities within and between countries [1].

Sexual health life in teenagers is of particular importance for the development of human capital and the reproduction of the population, ensuring well-being and productive growth, along with a good education. All of these will contribute to sustained socio-economic development improving the quality of life.

Although pregnancy should not be considered as a disease or burden, being a beautiful life event for most teenagers, it brings insecurity, problems, fear, and many questions. Pregnancy may arise problems at home with the parents, problems with the child’s father, and problems with society. The health issues of children born by teenagers could be major because, from a medical point of view, at this age, the woman is not anatomically and physiologically prepared to procreate without risk.

Statistics show that globally, every day, about 20,000 girls under the age of 18 become mothers, mainly in developing countries but not only. Of the total annual number of 7.3 million teenage mothers, those under the age of 15 represent two million. If these trends continue, the number of births from girls under the age of 15 could increase to three million by 2030 [2]. Births from teenage mothers account for 10% of all births in the world and 23% of maternal morbidity and mortality. Pregnancy at an early age is the leading cause of death in girls aged 15 to 19 worldwide; 90% of deaths occur in low-income countries and most can be prevented [2-3]. Among teenagers between 15 and 19 years old in Romania, annually, 5-10% get pregnant. The birth rate in these adolescents is constantly increasing. Romania ranks first in the European Union (EU) in the number of children born by mothers under the age of 15, representing a third of all EU cases, e.g., 676 in 2000. In fact, one in 10 births and one in 10 abortions were in 2016 at an early age, under 18 years of age (over 26,000 minors have become pregnant annually in Romania) [1].

Pregnancy in adolescence, at teens, especially in children under 15 years of age, is associated with higher maternal, obstetric, and perinatal risks than pregnancy in adulthood [4]. Therefore, pregnancy in adolescence is considered a pregnancy with a high risk of complications and requires special management [5]. Children born from teenagers, minor mothers, and mothers that are under 18 years of age, are both socially and medically vulnerable because they are often premature babies and come from pregnancies that have not been medically monitored [4-5].

The main objective of this research is to determine the outlined profile of the pregnant teenager and the medical implications associated with pregnancy in minor mothers in order to prevent neonatal complications and to identify the elements that could be acted upon in the future, for the prevention of the phenomenon in question.

This research was presented at the 1st International Electronic Conference on Medicine, 20-30 June 2021 (https://iecmd2021.sciforum.net/).

## Materials and methods

A cross-sectional study based on a 29-item questionnaire was conducted in 2019 and 2020 in Ploiești, Romania (Appendices). A total of 200 women who gave birth at the Hospital of Obstetrics and Gynecology between the ages of 13 and 42 voluntarily participated in this study with informed consent. The 200 women were grouped in two, namely, Group A, consisting of 100 teenagers, adolescents, and women under 18 years of age; and Group B, consisting of 100 women over 18 years of age.

For our study, we chose the Obstetrics and Gynecology Hospital in Ploiesti because the hospital is located in a country region that is about 60 km away from the Romanian capital, Bucharest, and is representative and similar to many other country hospitals in our country. The hospital serves not only the city of Ploiești but also the surrounding countryside.

The questionnaire was applied between November 1, 2019, and March 1, 2020. The reliability of the questionnaire was evaluated on a group of 20 volunteers by Cronbach's alpha coefficient and we obtained 0.82. The questionnaire consisted of 29 closed-ended questions, with the exception of questions related to name, surname, and age. It was administered by the same investigator in a face-to-face interview because not all participants could read and write.

The questionnaire contains general data (age, marital status, place of residence, number of children, occupation, level of education, ability to understand medical terms - health literacy - by applying the Short Assessment of Health Literacy (SAHL) test, aspects related to the family environment, parental education, parental occupation, housing characteristics, number of siblings). A frequently used measure in international research for the accurate measurement of health literacy is the SAHL test. A score between 0 and 14 suggests that the examinee has a low level of health knowledge [6-7].

In addition to the questionnaire, data on the obstetrical profile of the fertile woman and data on birth and newborn babies were collected. The resulting data were centralized in a database for both types of information.

Participation in the study was open to all women hospitalized during this period, up to 100 participants; for each minor pregnant woman interviewed, based on a voluntary option, another woman was selected for control Group B. These women were over 18 years old, hospitalized in the same ward, at the same period of time.

The group of 200 women represents 22.07% of the total number of births registered during the studied period.

Data analysis was performed using the program IBM SPSS Statistics 20.0 (IBM Corp. Armonk, NY) and Excel (Microsoft Corporation, Redmond, NY). We followed quantitative variables, processed according to distribution, dispersion indicators (minimum and maximum value, standard deviation), and central tendency indicators (mean, median), and in the case of qualitative variables, we calculated absolute and relative values. The variables were divided into two groups:

Socio-demographic characteristics: marital status, residence, education, level of knowledge about health, ethnicity, family status, and the number of rooms in the family home.

Information about pregnancy and birth: the number of pregnancy monitoring checks performed, number of medical investigations performed in pregnancy, number of hospitalizations performed in pregnancy, gestational age at birth, birth weight of newborns, and Apgar score of the newborn at birth.

Possible differences between sociodemographic and obstetric variables between lot A and lot B were analyzed using chi-square tests and Kruskal-Wallis. For the same situations, but in the case of continuous quantitative variables, we used the student's t-test. Statistically significant differences were those in which the p-value was less than 0.05 (p <0.05).

The study was conducted in compliance with the rules of medical ethics and was approved by the Ethics Commission of the hospital, "Ethics Committee of Scientific Research of the Hospital of Obstetrics and Gynecology, Ploiesti, Romania", final approval number: 2164 of 21 February 2019.

Patients agreed to participate by signing an informed consent form and agreed to the use of their data from the observation sheets and the results of the questionnaire. In the case of minor patients, consent was obtained from one of the parents.

## Results

The general characteristics of the groups are shown in Table 1. Teenage childbearing women from group A had a mean age of 16.56 ± 1.65. The highest frequency of births in this group is at 17 years of age. However, the number of births in very young adolescents (the group of 13-15 years old), which represents 28% of Group A is alarmingly high. The environment in which the studied teenagers developed could lead to pregnancy. In our study, we analyzed the data of the social status and we found that the childbearing teenager women came mainly from rural areas (Table 1). A significant percentage of teenage mothers are of Roma ethnicity, 76.47 (n = 39 from the total of 51 of Roma ethnicity childbearing women) and 92.85% (n = 13 from the total of 14) came from single-parent families (Table 1).

**Table 1:** Distribution of childbearing women according to age, place of living, occupation, education, ethnicity, level of knowledge about health, and standard of living of minor mothers

	Group A N=100	Group B N=100	P-value
Age			
Mean	16.56	26.28	
Standard deviation	1.65	6,31	
Median	17	26	
Number of rooms in the family home			
Mean	1.7	3.9	p < 0.00001
Standard deviation	0.98	1.2	
Median	1	3	
Level of knowledge about health (Health Literacy)			
Mean	11.28	16.04	p=0,00000
Standard deviation	4	2.63	
Median	12	18	
Place of living			
Rural	54	60	
Urban	46	40	
Occupation			
Household	67	52	p<0.0001
Employee	6	48	
Student	27	0	
Education			
No studies	12	0	p<0.00001
Primary cycle	27	8	
Gymnasium cycle	32	35	
Vocational school	22	27	
High school	7	22	
Higher education	0	7	
Postgraduate studies	0	1	
Ethnicity			
Romanian	58	88	p < 0.00001
Roma	39	12	
Other ethnicities	1	0	
Family status			
From families registered marriage	40	89	p = 0,011
From families whose parents are in cohabitation	35	10	
From families single-parent families	25	1	

Trying to analyze the economic level of the families from which the childbearing teenagers come, we found that in 15% (n = 15) of cases these young girls live in social housing, and in 9% (n = 9) of cases, the house where they live consists of a single room house compared to the control group, where social housing is encountered only in 5% (n = 5) of cases. We found that a high number of teenager childbearing women have parents with a low level of education (mean of classes completed by the minor mother = 6.78 ± 3.47, median 8, mean of classes completed by the minor’s mother = 6.25 ± 4.4, with median 8, mean of classes completed by the minor’s father = 6.65 ±4.11, with median 8). These adolescents are part of large families and live in a home with a fewer number of rooms compared to the number of family members (mean of members of minor´s family = 2.9 ±1.97, with median 2). We also noticed that the level of education of the teenager childbearing women and their families is low: the number of classes graduated by the minor mothers, as well as by their parents is lower than the number of mandatory classes in the current educational system in Romania. The analysis of the two groups shows that only 40 teenager mothers come from families whose parents have a registered marriage, the rest coming from families whose parents are in cohabitation (35 out of 100) or from single-parent families (25 out of 100); the difference is statistically significant compared to the control group (p = 0.011).

Analyzing the beginning of the sexual life of the respondents included in the study, we found that in 65 adolescents, the beginning of sexual life took place at an early age (under 14 years), in 12 cases at the age of 15-16 years, and in 17 cases at 17-18 years, the average age at which adolescents had their first sexual experience is 13.1 ± 1.63 years. In Group B, we didn’t assess the beginning of sexual life because only the present pregnancy was discussed. In the present study, in 38 cases, women of childbearing age reported sexual abuse in their medical history.

The application of the SAHL test to both groups showed a low level of health literacy in the group of adolescent childbearing women, Group A (Table 1). Evaluating the educational level and employment of childbearing women from the present study, we observed that, at the time of pregnancy, of the 84 adolescents with a low level of health literacy, 27 are students, six are employed, and the rest are neither students nor employed.

Regarding pregnancy monitoring, our study found statistically significant differences between the two studied groups (p < 0.001). For a childbearing woman, routine check-ups during pregnancy are very important. We noticed in the group of adolescent childbearing women, a mean of 3.52 ± 4.56 controls performed during all pregnancies, compared to 7.34 ± 5.75 controls in the control group. Studying the number of medical investigations performed during pregnancy, we found significant differences (p < 0.001): only a total of 116 childbearing women from both groups performed medical investigations during pregnancy and only 42 were teenagers from these 116. It is also noteworthy that out of a total of 95 childbearing women from both groups, who had hospitalizations during pregnancy, 56 were from the group of adolescent mothers compared to 39 from the control group (p = 0.016) (Table 2).

**Table 2:** Distribution of obstetrical data of pregnant women and newborns by weight and Apgar score

	Group A (N=100)	Group B (N=100)	P-value
Number of medical visits during pregnancy			
Mean	3.52	7.34	p < 0,001
Standard deviation	4.56	5.75	
Median	1	8	
Number of pregnant women that had medical investigations during pregnancy			
Yes	42	74	p < 0,001
No	58	26	
Number of pregnant women who had hospitalizations during pregnancy			
Yes	56	39	p = 0,007
No	44	61	
Gestational age at birth (weeks of pregnancy)			
Mean	36.88	38.41	p=0,0124
Standard deviation	2.13	1.57	
Median	36	39	
Birth weight of newborn babies			
<2000 g	1	1	
2000–2490 g	13	3	p < 0.00001
2500–2990 g	43	17	
3000–3490 g	31	48	
3500–3990 g	11	26	
4000–4490 g	1	4	
Over 4500 g	0	1	
Apgar score of newborn baby			
Mean	8.33	8.92	p = 0.00018
Standard deviation	1.12	0.68	
Median	8	9	

Regarding the diagnosis associated with pregnancy at the time of admission into hospital, it can be seen that in 17 cases, there were urogenital infections in the group of adolescent pregnant women compared to five cases in the control group, anemia occurred in five cases, early dysgravidia in three cases, preeclampsia in three cases, imminent miscarriage in 15 cases, placental insufficiency in 13 cases. In Group B, there were no diagnoses associated with pregnancy at the time of admission to a hospital.

According to the study, adolescent childbearing women gave birth to children with low weight for their gestational age; the difference was statistically significant (p < 0.00001) (Table 2). Regarding gestational age, in the case of teenage mothers, we had a mean of 36.88 ± 2.13 weeks of gestation, compared to the gestational age in the control group of 38.41 ± 1.57 weeks. The low birth rate in Group A is 14%, higher than in Group B (4%). The difference is statistically significant (Fisher's exact test: p=0.024).

Also, a higher incidence of newborn babies with lower Apgar at birth among teenage minor childbearing women was observed (p = 0.00018) (Table 2).

## Discussion

Analyzing the results of this study, we observed some common characteristics in the group of childbearing teenage mothers that define the profile of this category of pregnant women. Precarious socioeconomic conditions, greater vulnerability, low level of medical education, reduced addressability to medical services during pregnancy, a higher number of hospitalizations during pregnancy, health problems of the fetus, and Roma ethnicity are the main characteristic encountered.

Pregnancy in teenagers is strongly influenced by childhood experiences in the family and social environment, especially those related to unstable parenting or the model of early motherhood [8-10]. Pregnant adolescents who grew up in more disadvantaged families (rural areas, large families, did not live with both parents, poor mother’s education, non-prestigious occupation of the father, and Roma ethnicity), have higher risks for early pregnancy and early non-marital birth. Pregnancy in adolescents compromises women’s educational prospects and economic opportunities, being a marker of such conditions rather than a root cause [10].

This study highlights the precarious socioeconomic condition of minor mothers: although the pregnancy in minor mothers seems to be equally distributed between urban and rural areas as in the general population, minor childbearing women frequently neither work nor continue their studies; they come more frequently from single-parent families or from homes where their parents live in cohabitation.

The poor economic status of the families of the adolescents from the study increases their vulnerability. Their parents are either unemployed or do not have a stable job or in some cases, one of the parents is working abroad. Life without parental care and poor material conditions made these teenagers hope for marriage, and the birth of a child is often associated with a better perspective; they are like a lifeline, which will put an end to the problems they face.

Due to pregnancy, most adolescent mothers drop out of school, unable to receive the education they need [1]. According to data from the literature, the level of education and welfare status of women has a significant impact on pregnancy in adolescents [11]. In the analyzed group of minor mothers, 12% (n = 12) are without studies at all and only 7% (n = 7) have completed or are in the process of completing high school.

Numerous studies have evaluated the impact of pregnancy and childbirth on adolescents from a medical, psychological, and socio-economic point of view [11-12]. The results of these studies show how much adolescents rely on the support and backing of their family and community. Thus, adolescents who benefit from these advantages are more likely to continue their studies and get employed [11]. The increased risk of giving birth to a low birthweight baby, in the case of adolescents, is determined by social factors (poor prenatal evidence, poverty) and behavioral factors (smoking, substance abuse) and less by biological ones [13].

Many adolescents begin their sexual life earlier and, in a significant proportion, they are victims of sexual abuse. Our study shows that 38% (n = 38) of minor mothers reported that the pregnancy occurred as a result of sexual abuse. Unfortunately, the number may be, in reality, higher, as a considerable number of teenagers avoid discussing this topic out of fear or out of personal reasons. Analyzing the beginning of the sexual life of the respondents included in the study, we found that in 65 adolescents, the beginning of sexual life took place under 14 years, in 12 cases at the age of 15-16 years, and in 17 cases at 17-18 years. Thus, the probability of starting sexual life earlier than the age of 16 years old is high; the mean age at which adolescent girls said they had their first sexual experience is 13.1 ± 1.63 years.

The result of our study agrees with other research results that have shown that the first sexual experience for most adolescents is related to drunkenness, drug use, spending nights away from home, or is the consequence of sexual abuse and hidden violence [4,14]. Pregnancy is a special stage for any woman; hormonal changes during this period increase the sensitivity of the pregnant woman to internal and external stimuli, increasing vulnerability to stress and unforeseen events.

At the same time, our study demonstrates the low level of medical education. From the analysis of the answers given to the SAHL test by the adolescents participating in the study, it can be seen that they do not know the notion of condoms or viruses. Illiteracy in adolescence interacts significantly with pregnancy and can lead to complications with significant effects on maternal health, both immediately and after pregnancy. Pregnancy and childbirth in adolescents are complex social problems, with medical, social, and economic consequences, and the early age of the onset of sexual life and the increased risk of pregnancy resulting in complications is a cause for concern. All of these could be explained by the lack of information in the group of teenage mothers about the damage of early motherhood and contraceptive methods [15].

Most teenage mothers are Roma. The explanation would be the fact that the values ​​according to which they are guided differ enormously from the values of an ordinary Romanian woman. Their nationalism is still firmly rooted in the Roma tradition, which translates into respect for ancient traditions, respect for Gypsy culture, and customs known to ancient ancestors. Roma families are characterized by the following features: early marriages to young age, no legal basis, a high number of children, and low divorce. Marriage to Roma is still, in many cases, concluded only according to the rules of the community to which it belongs, without being legalized.

The present study was conducted in a country where the licensed midwifery profession appeared in 2011. In Romania, there are currently 1000 licensed midwives, but they cannot be integrated into the care system due to the gap in legislation, according to a statement from the Ministry of Health [16] although the development of this network could facilitate some of the problems of pregnancy in minors. According to a study by the State of the World’s Midwifery, well-trained midwives could help prevent about two-thirds of all maternal and newborn deaths. They could also provide 87% of all essential sexual, reproductive, maternal, and neonatal health services. However, only 42 percent of midwives work in the 73 countries where more than 90% of all maternal and neonatal deaths occur [12]. Although in the countries of the European Union the assistance provided by midwives is a natural practice, in Romania, there are still no rules of practice, guidelines, and professional protocols that clearly regulate this profession by harmonizing with European legislation [16]. The midwife may play an essential role in the rural communities, especially in the Roma communities or in other disadvantaged communities, the midwife can identify the minor pregnant woman, report the teenage pregnancy cases to the competent authorities, supervise the pregnant woman, counsel for the prevention of unwanted pregnancies in minors, and detect the medical and social needs to obtain medical and social benefits.

The lack of health education and knowledge of medical terms is evident in the group of teenage childbearing women. Accurate measurement of health literacy is essential to improve the accessibility and effectiveness of health care and prevention. One measure frequently applied in international research is the SAHL test. A score between 0 and 14 suggests that the examinee has a low level of health literacy [6-7]. The application of the SAHL test to both groups showed a low level of health literacy in the group of adolescent childbearing women vs. the control group.

The level of education, economic precariousness, and social life could be the explanation for the reduced addressability to medical services during pregnancy. Adolescent pregnant women performed statistically significantly fewer controls during pregnancy, benefited from fewer screening tests, and had more hospitalizations during pregnancy. Research shows that pregnant adolescents are less likely to receive quality prenatal care, often late in the third trimester of pregnancy, or limited to childbirth care [17]. Studies report that one-third of pregnant adolescents receive insufficient prenatal care, and their children are at increased risk of developing health problems or prolonged hospitalization compared to children of adult women [18]. Thus, in the case of teenage pregnant women who had hospitalizations during pregnancy, the most common diagnoses are urogenital infections, imminent miscarriage, and placental insufficiency. The high frequency of these complications could be explained by the immaturity of the maternal organism.

Regarding the onset of childbirth, it was found that a considerable percentage of the group of adolescents had premature rupture of membranes (n = 56 compared to n = 23), a situation that could be explained by the more frequent presence of sexually transmitted infections. The high prevalence of these infections [19] is also due to a low level of knowledge about sexually transmitted infections in adolescents aged 14-19 years compared to young people aged 20-24 [19-20]. Sexually transmitted infections in pregnancy are complicated, regardless of the age of the mother with premature birth, chorioamnionitis, puerperal infections, etc. Adolescents have been shown to have sexually transmitted infections that increase the risk of premature rupture of membranes [20]. In our study, most adolescents did not go to the doctor during pregnancy, so they did not benefit from methods to detect these infections and consequently adequate treatment. In the case of hospitalization during pregnancy, the predominant diagnosis was urinary tract infections supporting the idea of sexually transmitted infections.

The socio-economic and medical factors listed above have repercussions on the fetus. The data obtained in our study show that in Group A, there is an increased frequency of babies with low birth weight and a lower mean Apgar score at birth compared to the control group. Data from the literature suggest that newborn babies from adolescent mothers have a more difficult postnatal adaptation [21].

In our study, of the total number of babies born from minor mothers, 14%, (n = 14) weighed less than 2490 g compared to only 4% (n = 4) of newborn babies in the control group. Data from the literature suggest that neonatal prematurity is related to the mother’s age. The low level of estrogenic and progesterone secretion in the preconception period determines the formation of placental insufficiency, hypoxia, and intrauterine growth restriction in the fetus. Low birth weight suggests the need for access to specialized medical care for pregnant women. These complications can not only be correlated with the low number of prenatal visits, late initiation of prenatal care, inadequate prenatal care but also other factors such as race, marital status, low level of schooling, and poverty. In our study, we found a mean of 3.52 ± 4.56 medical controls performed during pregnancy and a median of 1 in Group A compared with 7.34 ± 5.75 medical controls in group B, with a median of 8.

It has also been found that there is a direct link between low birth weight and the young age of the mother, which has been explained by the fact that adolescents have a shorter cervix and a smaller uterine volume [22]. Moreover, due to their precarious social status, adolescent mothers may have a lower concentration of glycine or other amino acids for intrauterine growth and development; these nutritional deficiencies could lead to intrauterine growth retardation and lower birth weight [23]. Biologically, cervical and uterine vascularity is not fully developed in pregnant adolescents. Hemostatic reactions in adolescents are unstable, which aggravates the prognosis for mother and fetus and determines the incidence of hemorrhagic disorders in the immediate postpartum [24]. Poor health literacy may also show in low vaccination education [24-30]. The evolution of pregnancy and childbirth in adolescents has distinctive features because it takes place against an unfavorable background: biological immaturity of the body and underdeveloped neuroendocrine and immune systems. Pathological changes in the mother’s body lead to the decreased adaptive capacity of the fetus and disturb the balance in the mother system, placenta, and fetus, as illustrated in our study by the lower birth weight and significantly lower Apgar score in children born to minor mothers.

## Conclusions

In Romania, most teenagers that became mothers at an early age have Roma origin. The level of education of the young mother is linked with the occurrence of early pregnancy. Therefore, we noticed early school leaving, unemployment, and losing interest in acquiring a profession, all these making the teenager grow into an adult that cannot support either herself or her child. The teenagers that become pregnant between 13-15 years come from poor families that are unable to provide adequate medical supervision, with significant consequences for the health of the mother and child, which most of the time is a premature baby. The phenomenon is cyclical; it reproduces within the same family, from one generation to another, with all the procession of economic, social, and health precariousness that accompanies it.

The midwife may play an essential role in rural communities, especially in the Roma communities or in other disadvantaged communities. The midwife may be involved in educational programs intended for minor pregnant women to obtain medical and social benefits. Better management of educational policies and a multidisciplinary approach is needed. Future research may establish if the information needed by the teenagers should be taught in schools, when is the most appropriate time, and who would be the best person to explain it.

## Appendices

**Table 3:** Questionnaire

Nr. question	Question
1	Age
2	Place of residence
3	Ethnicity
4	Occupation
5	Level of education
6	Ability to understand medical terms - health literacy - by applying the Sahl test
7	Marital status
8	Marital status of parents
9	Parental education
10	Parental occupation
11	Number of siblings
12	Housing characteristics
13	Number of children
14	What was the age of pregnancy at the first doctor's consultation?
15	How many times have you been to the doctor during pregnancy?
16	Did you investigate during pregnancy?
17	Did you have any tests during pregnancy?
18	Which sources do you think you can get information about pregnancy?
19	Who did you talk about pregnancy for the first time?
	What did the person you were talking to advise you?
20	Were you hospitalized during pregnancy?
21	What was the diagnosis? Why?
22	How old were you when you first had sex?
23	Was it a desired experience?
24	Did you want this child?
25	Did you use the contraceptive method?
26	What contraceptive methods do you know?
27	What sexually transmitted diseases do you know?
28	What is your relationship with the child's father?
29	How do you intend to raise your child?
